# Repurposing existing drugs for new uses: a cohort study of the frequency of FDA-granted new indication exclusivities since 1997

**DOI:** 10.1186/s40545-020-00282-8

**Published:** 2021-01-04

**Authors:** Babak Sahragardjoonegani, Reed F. Beall, Aaron S. Kesselheim, Aidan Hollis

**Affiliations:** 1 Department of Economics, University of Calgary, 527 Campus Place N.W., Calgary, AB T2N 4Z6 Canada; 2 Department of Community Health Sciences, Cumming School of Medicine and O’Brien Institute for Public Health, University of Calgary, 3280 Hospital Drive NW, Calgary, AB T2N 4Z6 Canada; 3 Program on Regulation, Therapeutics, and Law (PORTAL), Division of Pharmacoepidemiology and Pharmacoeconomics, Department of Medicine, Brigham and Women’s Hospital / Harvard Medical School, 1620 Tremont St., Suite 3030, Boston, MA 02120 USA

## Abstract

**Background:**

Drug repurposing (i.e., finding novel uses for existing drugs) is essential for maximizing medicines’ therapeutic utility, but obtaining regulatory approval for new indications is costly. Policymakers have therefore created temporary indication-specific market exclusivities to incentivize drug innovators to run new clinical investigations. The effectiveness of these exclusivities is poorly understood.

**Objective:**

To determine whether generic entry impacts the probability of new indication additions.

**Methods:**

For a cohort of all new small-molecule drugs approved by the FDA between July 1997 and May 2020, we tracked new indications added for the subset of drugs that experienced generic entry during the observation period and then analyzed how the probability of a new indication changed with the number of years since/to generic entry.

**Results:**

Of the 197 new drugs that subsequently experienced generic entry, only 64 (32%) had at least one new indication added. The probability of a new indication addition peaked above 4% between 7 and 8 years prior to generic entry and then to dropped to near zero 15 years after FDA approval. We show that the limited duration of exclusivity reduces the number of secondary indications significantly.

**Conclusion:**

Status quo for most drug innovators is creating novel one-indication products. Despite indication-specific exclusivities, the imminence of generic entry still has a detectable impact on reducing the chances of new indication additions. There is much room for improvement when it comes to incentivizing clinical investigations for new uses and unlocking existing medicines’ full therapeutic potential.

## Key points

- Drug repurposing is finding novel uses for existing drugs.
- Indication-specific market exclusivities have been used to incentivize drug innovators to conduct the clinical trials preferred by drug regulators for label changes, but these are difficult to enforce because generic equivalents may still be approved and used off-label.
- Our study finds that, despite indication-specific exclusivities, no new indications are added after FDA approval for two-thirds of drugs and that the probability of new indication additions drops to near zero after generic entry occurs.
- As status quo for most drug innovators appears is creating novel one-indication products, there is much room for improving incentivizes for clinical investigations for new uses and for maximizing existing medicines’ full therapeutic potential.

## Background

Drug repurposing (i.e., using an existing drug for a new use) is essential for making the most of our medical armamentarium. Many drugs have accrued new indications over a period of years: adalimumab (Humira) was approved by the FDA for sale in 2002 with one indication and now has 10; onabotulinumtoxinA (Botox) was approved in 1989 with two indications and now has 9. Berndt et al. examine the history of uses of histamine-2 antagonists, proton-pump inhibitors, and selective-serotonin reuptake inhibitors and find that about 75% of their use was ultimately for uses other than their first-approved indications [1]. Drug repurposing has become one of the key tools in the search for medicines in the early fight against Covid-19 [2, 3], with existing products remdesivir and dexamethasone both being found to show improvements in clinical endpoints among hospitalized patients [4].

The timing of new indication innovation varies considerably between drugs. For many drugs, repurposing happens during the development process. For example, remdesivir, initially explored as a treatment for hepatitis C virus infection, was tested unsuccessfully against Ebola, and eventually demonstrated efficacy in reducing the length of hospitalization for patients with COVID-19. While our focus in this paper is on new indications added after a primary indication has been approved, the fact that drugs may be tested for multiple indications before receiving any approval is testimony to the fact that researchers may not know all the possible uses for a drug when it is developed. Sometimes new uses are explored using high-throughput screening, genomic, transcriptomic or proteomic analysis, and analysis of administrative health data [5]. For other drugs, including famously sildenafil (Viagra), indications that become the basis for repurposing are discovered fortuitously through patient experience.

When new indications are discovered for already-approved drugs based on trials showing some evidence for effectiveness, physicians may then begin to use a drug for that purpose “off-label” [6–12]. However, many off-label uses are not based on high-quality clinical trials and in general any studies supporting an off-label use have not been reviewed by an expert regulator, or have been reviewed and found not to support a new indication, so they may be biased or may need to be weighed against other negative trials. As a result, the drug being used off-label may be ineffective or even unsafe, a point made clear by the recent widespread use of hydroxychloroquine for Covid-19 [13]. Off-label use is particularly common in children. A recent study of ambulatory pediatric visits in the US found that that 38% of prescriptions were off-label, with the majority being off-label by reason of indication rather than age [14–17]. The ubiquity of off-label use suggests that there is a need for more clinical trials to support new indications for approved drugs.

Obtaining regulatory approval for new indications may be costly. To address this challenge, policymakers in the US and in some other countries have designed incentive programs that offer manufacturers that obtain a new indication for an existing drug a temporary market exclusivity in the use of the drug for the newly approved indication [18]. These exclusivities last for 7 years for drugs intended to treat rare diseases and 3 years for more common conditions in the US.

These exclusivities, however, are difficult to enforce, particularly if there is existing competition from generic versions of the same drug already available for ongoing off-label use by prescribers. Without the revenue that would arise from monopoly pricing during market exclusivity, manufacturers may not have sufficient market-driven incentive to make the upfront investment in clinical trials preferred by regulators to validate these new uses [19, 20]. In the cases of adalimumab and onabotulinumtoxinA, for example, numerous indications were tested after FDA approval during long exclusivity periods free of generic competition.

To investigate the possible impact of generic competition upon post-approval indication innovation, we sought to investigate the relationship between the timing of generic entry and the number of new post-approval indications added per drug. Our hypothesis was that once generic competition is present, fewer new indications are FDA-approved and that as generic entry approaches, the probability of new indications being added would diminish. As such, we expected that the highest probability of new indications would be in the years soon after drug approval because it would provide the manufacturer with the longest period of exclusivity during which to reap the benefits and that most exclusivities associated with new indications will expire before generic entry (since generic entry typically occurs about 12–14 years after FDA approval [21, 22]).

## Methods

### Design

For a cohort of all new small-molecule drugs (i.e., New Molecular Entities [NMEs]) approved by the FDA between July 1997 and May 2020 (23.4 years), we tracked new indication exclusivities granted, and for the subset of drugs that experienced generic entry during the observation period, we analyzed the relationship between the number of years since/to generic entry and the number of new indications granted.

### Data sources

Our chief data source was electronic archives of the FDA’s Approved Drug Products with Therapeutic Equivalence Evaluations (the “Orange Book”) covering July 1997 through May 2020 [23]. All new indications that have been added on the basis of a new clinical investigation by the drug’s manufacturer receive an exclusivity. These datasets included the timing and type of new indication exclusivity (i.e., a 3-year New Indication exclusivity or a 7-year Orphan Drug Exclusivity) as well as which products the exclusivities were protecting. The Orange Book also provides all small-molecule drugs’ active ingredients, trade names, manufacturers, FDA approval dates, and whether the products are brand name or generic. To distinguish which brand-name drugs were NMEs, we linked to the Drugs@FDA Data Files [24] so that all subsequently approved brand-name drugs (i.e., new formulations) could be grouped with their parent products.

### New variables

To study the association between the timing of generic entry and the frequency of a new indication, we created a variable (i.e., “Age”) for the NME’s ages during each observation year by calculating the number of years since their respective FDA approval dates. We also generated variables with Age raised to the power 2, 3, and 4 to allow for a non-linear relationship between age and the frequency of new indications. For NMEs with an equivalent generic drug approved during the observation period, we calculated the number of years to/since the first FDA approval of a generic equivalent. We then constructed dummy variables categorizing time to generic as being more than 10 years, 5–10 years away, 0–5 years away, or post-generic entry. Using these variables, each outcome (i.e., the number of new indications added) could be observed in years according to the new drug’s FDA approval date and in time period since/to the first generic approval. Since our objective was to focus upon new indications added during the post-approval period and to isolate the effect of generic entry, we excluded any new indications added at the time of FDA approval (i.e., AGE = 0) as well as drugs that had no generic equivalents by May 2020. Finally, for each year observed since NME approval and to/since generic entry, we observed whether there was either zero or at least one new indication. There was one observation for each year following the approval of each parent drug until 2020, for a total of 3154 observations.

We also categorized drugs by disease category in which the drug was first introduced, and calculated the proportion of drugs within each disease category. (We applied the MeSH disease categories from the National Library of Medicine.)

### Analysis

We first report basic descriptive statistics of interest, including the proportion of drugs with one or more post-approval indications added during the observation period, the proportions of new indications added for more common versus rare diseases, and the proportion of new indication exclusivities that expired before versus after generic entry.

Given that generic entry occurred at different times for different drugs (Additional file 1: Fig. 1), the variation in a new drug’s period on the market (“age”) at generic entry can be used to disentangle the effects of age and generic entry on the probability of new indication development. We used the following logistic regression model to study the effect of the first generic entry timing on the possibility of having a second indication controlling for the drug’s age:

$$\ln \left( {\frac{p}{1 - p}} \right) = \alpha_{1} {\text{Age}} + \alpha_{2} {\text{Age}}^{2} + \alpha_{3} {\text{Age}}^{3} + \alpha_{4} {\text{Age}}^{4} + \beta_{1} G_{{{\text{neg}}}} + \beta_{2} G_{0,5} + \beta_{3} G_{5,10} + \varepsilon ,$$

where $p$ is the probability of having a new indication; Age is observation year minus the new drug’s FDA approval date; and *G* is the years until generic entry (with ${G}_{\text{neg}}=1$ as observations following generic entry, ${G}_{\mathrm{0,5}}=1$ during the 5 years before generic entry, and ${G}_{\mathrm{5,10}}=1$ from 5 to 10 years before generic entry). The omitted category is all years more than 10 years before generic entry. Thus, the regression coefficients for the time to generic variables (*G*) in the model represent the predicted change in the logarithm of the odds ratio for each time-to-generic-entry category, compared to when generic entry is more than 10 years in the future. We use the polynomial values of Age up to ${\text{Age}}^{4}$ to ensure that we capture all the variation caused by years since the drug’s first approval.

Finally, while controlling for age, we estimated the counterfactual number of new indications, assuming the influence of generic entry were the same as when generic entry had already occurred, as when it is 0–5 years in the future, and as when it is 5–10 years into the future. To calculate the counterfactual number of second indications, we used the estimated results and replaced ${\beta }_{1}$ and ${\beta }_{2}$ with the estimated value of ${\beta }_{3}$ (i.e., generic entry is not to occur for another 5–10 years), adjusting appropriately for the frequency of observations with multiple new indications.

## Results

During the 23-year observation period, 197 new drugs were approved and subsequently experienced generic entry. Of these, 64 (32%) were issued at least one post-approval new indication. As shown in Additional file 2: Table 3, 163 of these drugs had only one indication at the time of approval; 22 had two indications, and 12 had three or more indications.

### Incidence of post-approval new indications and timing of their associated exclusivity expirations

There were 116 post-approval new indications added during the observation period for the 64 genericized drugs (Table 1). About one-quarter (27/116, 23%) new indications expired after generic entry had occurred. Expiration after generic entry was more common for the longer-lasting rare disease indications with 14 of 18 (78%), expiring an average of 4 years after. Expiration after generic entry was less frequent among common conditions with 13 of 98 (13%), expiring an average of 85 days after.

**Table 1:** Count of post-approval new indications per drug

Count of new indications per drug	3-year new indication exclusivities	7-year new rare disease indication exclusivities	Total
Drugs with 1 new indication	29 (83%)	6 (17%)	35 (100%)
Drugs with 2 new indications	32 (84%)	6 (16%)	38 (100%)
Drugs with 3 or more new indications	37 (86%)	6 (33%)	43 (100%)
Total	98 (84%)	18 (16%)	116 (100%)

Over the 23-year observation period, the cohort of drugs for which generic entry was observed had a total of 116 new indications added during the post-approval period collectively. The majority were for common diseases, rather than for rare diseases, despite the difference in their duration (i.e., 3 versus 7 years, respectively). 70% (38 + 43 = 81) of the new indications were not the first new indication added for that same drug (i.e., they were the second, third, or even the fourth new indication added during the post market period). This may imply that some drug innovators are much more active than others in seeking out new indications

### Probability of new indication exclusivities by NME age

**Fig. 1:**
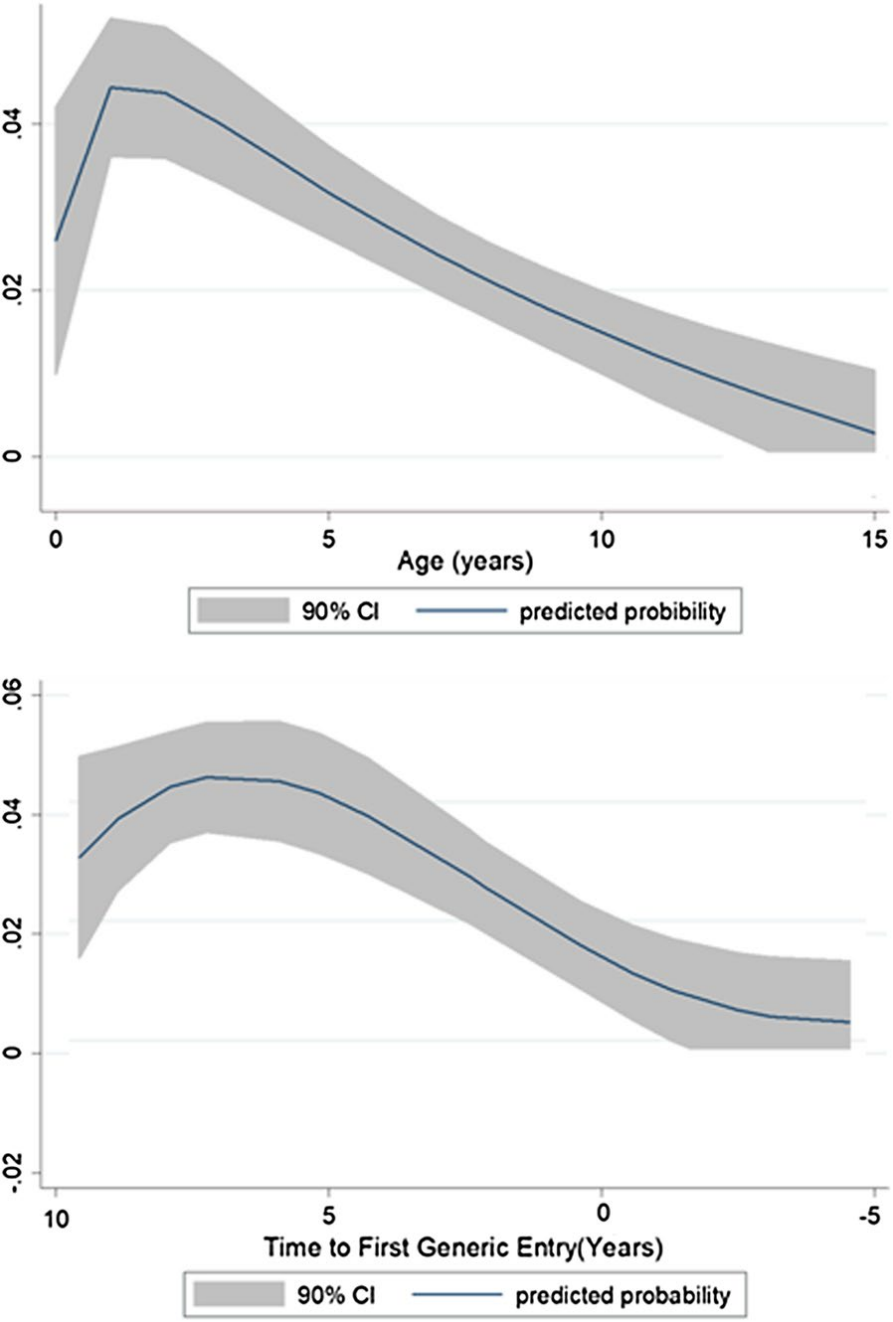
Probability of new indication exclusivity granted by FDA for drugs approved, 1997–2020. The probability of a new indication addition is highest when NMEs have been relatively recently (within 1–2 years) and when generic entry is around 7–8 years into the future. The probability is lowest when an NME has been approved for more than 15 years and when generic entry has already occurred. “Age (in years)” = number of years since FDA approval

The probability that new indications would be developed changed according the age of the new drug. The probability peaked above 4% between 1–2 years after FDA approval and then dropped off (Fig. 1). By 15 years after FDA approval, the probability dropped to less than 1%, or about one-quarter of the peak.

### Probability of new indication exclusivities by year until/since generic entry

Similarly, the probability that a new indication would be developed also changed according to the number of years until the new drug experienced generic entry. The probability of a new indication peaked above 4% between 7 and 8 years prior to generic and then dropped off (Fig. 1). By 15 years after FDA approval, the probability dropped to near zero.

### Counterfactual impact on the probability of new indication exclusivities controlling for NME age

**Fig. 2:**
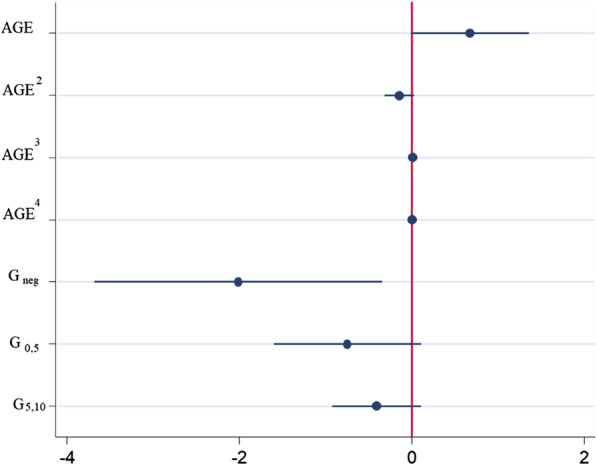
Estimated regression coefficients. The imminence of generic entry negatively impacts the chances of a new indication being developed for an NME. Holding the effect of age constant, generic entry’s influence upon reducing the chances of a new indication addition is strongest when generic has already occurred and weakest when generic entry is still 5–10 years into the future

When controlling for a new drug’s time on the market, regression results show that the probability of receiving a new indication exclusivity was independently conditional on the timing of generic entry (Fig. 2, Additional file 2: Table 1). The probability of new indication exclusivity dropped consistently from 5–10 years before generic entry, to 0–5 years before generic entry, and then especially after generic entry had already occurred.

### Predicted probabilities of new indications if the impact of generic entry is applied to all drugs

**Fig. 3:**
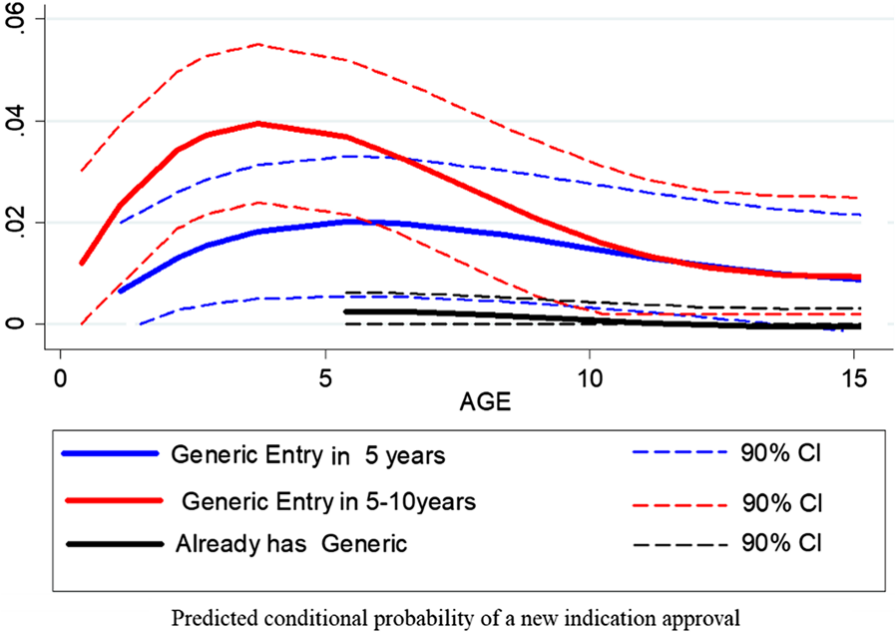
Predicted conditional probability of a new indication exclusivity approval. When we applied the strongest observed influence of generic entry (i.e., generic entry had already occurred [${\beta }_{1}$]) to all NMEs, shown in black is our model’s predicted probability of new indications additions, which is near zero, regardless of the age of the drug. Note that by law, generic entry cannot occur in the United States prior to 5 years after FDA approval when the product in question is designated as a small-molecule NME, and therefore, the black line begins only after 5 years accordingly. When we applied the weakest observed influence of generic entry (i.e., generic entry is 5–10 years into the future [${\beta }_{3}$]) to all NMEs, shown in red is our model’s predicted probability of new indications additions, which is substantially higher, regardless of the age of the drug

In the counterfactual case that all drugs had the same probability of a new indication as when generic had already occurred, our results show that the chance of a new indication exclusivity was nearly zero, regardless of the new drug’s time on the market (Fig. 3). By contrast, in the counterfactual case that all drugs had the same probability as when generic entry is 5–10 years into the future, the probability peaked at nearly 4% after around 4 years after FDA approval and before declining and levelling out to around 1% after around 15 years after FDA approval. Our model predicts that if the effect of generic entry could be removed, the expected number of new indications for our cohort of NMEs would have been 134 rather than the 116 which occurred in actuality—in other words, a 16% increase was predicted (Table 2).

**Table 2:** Real and counterfactual number of new indications additions

Time to generic*	Probability of having a new indication		Number of new indications	
	Real (%)	Counterfactual (%)	Real	Counterfactual
*G*_0,5_ = 1	1.07	1.29	34	41
*G*_5,10_ = 1	1.64	1.64	45	45
*G*_10+_ = 1	1.80	1.80	34	34
*G*_neg_ = 1	0.08	0.37	3	14
Total			116	134

${G}_{neg}$ indicates observations following generic entry; ${G}_{\mathrm{0,5}}$ observations when generic entry is within 5 years, ${G}_{\mathrm{5,10}}$ observations when generic entry is within 5 to 10 years from 5 to 10 years; ${G}_{10+}$ observations when generic entry is more than 10 years away

### Therapeutic area differences

The proportion of drugs which received a secondary indication following approval varied noticeably across disease categories, as shown in Table 3. Secondary indications were most common for drugs categorized as treating neoplasms and skin and connective tissue disease.

**Table 3:** Frequency of secondary indications by disease area

Disease group	Number of associated approved drugs	Number of drugs with post-approval indication	Percent with post-approval indication (%)
Neoplasms	22	12	54
Skin and connective tissue disease	19	9	48
Digestive system diseases	12	5	41
Musculoskeletal diseases	10	4	40
Endocrine system diseases	8	3	38
Respiratory tract diseases	9	3	33
Nutritional and metabolic diseases	18	6	33
Pathological conditions, signs and symptoms	7	2	29
Hemic and lymphatic disease	11	3	27
Nervous system disease	52	14	26
Infections	27	7	25
Cardiovascular disease	28	7	25
Immune system diseases	13	3	23
Female urogenital diseases and pregnancy complications	14	3	21
Chemically induced disorders	5	2	20
Male urogenital diseases	17	3	18
Stomatognathic diseases	1	0	0
Eye diseases	5	0	0
Congenital, hereditary diseases, and neonatal and abnormalities	8	0	0
Wounds and injuries	2	0	0

Drugs indications are extracted from *FDA* database. Each indication is matched with related *USA National Library of Medicine* Drugs Category. Data are drugs approved by FDA after 1997

## Discussion

Our analysis shows that for two-thirds of all new drugs developed, no new indications are added during the post-approval period by drugs’ manufacturers. New uses are mostly added soon after FDA approval and long before generic entry, particularly for new indications for more common conditions (as opposed to rare diseases). Generic entry’s effect on reducing new indication innovation activity makes a detectable difference. Together, these findings may imply that there is much potential for improvement when it comes to providing incentives for clinical investigations that lead to regulatory authorization of new uses of approved drugs.

Our finding that two-thirds of drugs have no new indications added during the post-approval period can be explained in two (non-exclusive) ways: either those drugs are intrinsically highly specific to a disease area or the incentive to invest in clinical trials to obtain new indications is weak. To the extent that it is the latter reason, the failure to make full use of drug repurposing opportunities reduces the value of existing medicines: it reduces the effectiveness of our therapeutic toolkit and also puts patients at risk. Previous studies have demonstrated that once a drug is approved, off-label use is widespread (as high as 46% of all prescribing for some types of medications, such as cardiac medications and anticonvulsants) and that most (73%) off-label uses have little or no scientific basis [7]. In other words, a substantial share of prescribing is not validated by the FDA and is based on weak data. Historically, drug representatives have not been allowed to advertise off-label uses of their products to prescribers, although over the past two decades nearly all pharmaceutical manufacturers have been investigated for violating this rule [25]. Further, a number of practice guidelines and practice compendia, such as in psychiatry, include off-label uses [9]. It would be preferable if common uses of all drugs could be validated by the neutral experts at government regulators like the FDA based on high-quality clinical trials.

Most new indication market exclusivities that have been issued by the FDA expire before generic entry based on the original drug approval. This occurs because a drug’s patents last further than most post-approval exclusivities granted for new indications [26, 26]. Thus, these exclusivities seldom play a practical role in market protection. To increase incentives to obtain secondary indications, the 3-year exclusivity could be lengthened, or the exclusivity could be extended to all patents and other exclusivities that exist for that same product. This is currently done for clinical trials to test existing indications for adults in pediatric populations (i.e., a new patient population)—a 6-month extension is given to all patents and exclusivities [27]. This policy has motivated a large number of pediatric trials, many extended indications and a small number of new indications, though at a high cost: the return on investment for drug makers has been shown to be highly lucrative [28]. Shifting to an exclusivity and patent extension model (even by a period of 3 months, rather than the current 3-year exclusivity that expires before it can protect the market in practice) would have an impact, although the therapeutic benefits would have to be weighed against the financial costs of extended exclusivity.

Other policy alternatives that are non-exclusivity-based are also possible. For example, priority review vouchers which can be sold or used to expedite FDA receive of new drugs (as is currently being done to incentivize research and development of neglected tropical diseases [29]), tax breaks (as is currently being done to incentivize treatments for rare diseases [26]), or greater upfront government funding are some examples which each have their own strengths and limitations. For example, priority review vouchers have attracted considerable criticism [30]. An important advantage of non-exclusivity-based incentives is that the timing of generic entry is unaffected, which is important for medicine accessibility, affordability, and public health impact.

Our study has certain limitations. Most importantly, the counterfactual estimate does not account for the fact that even when generic entry is distant, there may still be a disincentive to invest in the clinical trials necessary to establish a new indication, given that generic entry will eventually reduce the total possible sales of the drug in all indications. We have not been able to include biologics in this analysis, despite their growing importance, because of the small number of drugs with biosimilar competition. Moreover, the regulatory framework around biologics differs in numerous ways, which makes comparisons treacherous.

## Conclusion

For two-thirds of new drugs approved in recent years by the FDA, drug manufacturers added no new indications during the post-approval period, suggesting that even when an off-label use becomes common, all too often no one invests in securing FDA approval for it. One way to address this issue would be to redesign current incentive structures such that firms can profit from investments into new clinical trials that meet FDA standards and could be submitted for inclusion on the official drug labeling. Such a move would help weed out secondary uses based on solid versus weak data and would help improve the dissemination of evidence-based prescribing practices, for the benefit of patients.

## Supplementary Information

**Additional file 1:** Figure A-1. Time between brand and first generic entry.

**Additional file 2:** Appendices.

## Data Availability

This study is not reporting data from a trial. All raw data are publicly available from the United States Food and Drug Administration**.**

## Patient involvement

This study required no direct patient or public involvement.
