# Supplementary figures and images for: Repurposing existing drugs for new uses: a cohort study of the frequency of FDA-granted new indication exclusivities since 1997
Source: J Pharm Policy Pract. 2021 Jan 4;14:3. doi: 10.1186/s40545-020-00282-8 (PMC7780607; doi:10.1186/s40545-020-00282-8)

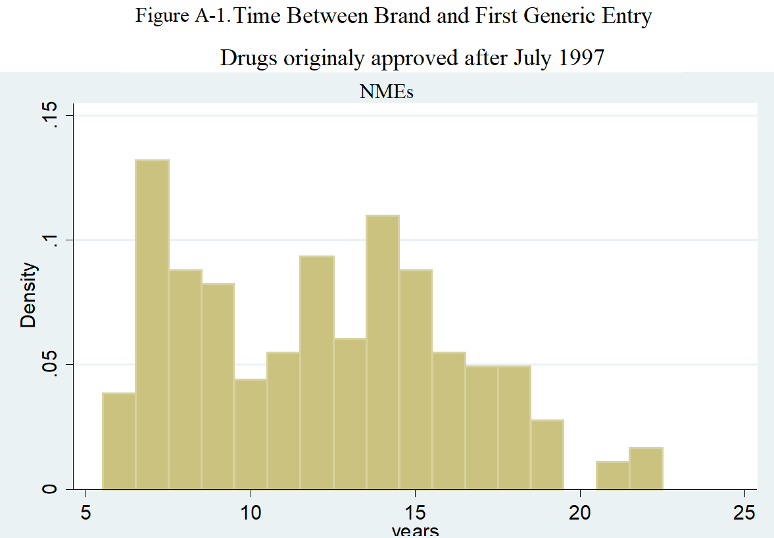

Supplement: Supplementary file 1 — Additional file 1: Figure A-1. Time between brand and first generic entry. [file 40545_2020_282_MOESM1_ESM.png]
