# Supplementary material for: Repurposing existing drugs for new uses: a cohort study of the frequency of FDA-granted new indication exclusivities since 1997
Source: J Pharm Policy Pract. 2021 Jan 4;14:3. doi: 10.1186/s40545-020-00282-8 (PMC7780607; doi:10.1186/s40545-020-00282-8)
Supplement: Supplementary file 2 — Additional file 2: Appendices. [file 40545_2020_282_MOESM2_ESM.docx]

# **APPENDICES**

**APPENDIX TABLE A1. Estimated coefficients for logistic regression**

| **Appendix Table 1. Estimated coefficients for logistic regression** | |
| --- | --- |
| Age | 0.674 |
|  | 0.35 |
| Age^2^ | -0.144 |
|  | 0.08 |
| Age^3^ | 0.009 |
|  | 0.01 |
| Age^4^ | -0.0002 |
|  | 0.0002 |
| G_neg_ | -2.012 |
|  | 0.85 |
| G0,5 | -0.745 |
|  | 0.43 |
| G5,10 | -0.306 |
|  | 0.27 |
| Observations | 3154 |

*Legend: Age is observation year minus the NME’s FDA approval date. Polynomial values of Age were used up to* ${Age}^{4}$ *in order to ensure that all the variation of caused by years since the drug’s first approval were captured. G is the years until generic entry (with* $G_{neg}=1$ *as observations following generic entry,* $G_{0,5}=1$ *during the five years before generic entry, and* $G_{5,10}=1$ *from 5 to 10 years before generic entry).*

**APPENDIX FIGURE A1. Time between brand approval and first generic entry**

*Legend: There was wide variability in the age of an NME when generic entry occurred. As this distribution differed from the age of an NME when new indications were added, it was possible to disentangle the effects of age and generic entry upon the probably of new indication development separately. There were no drugs with generic entry prior to 5 years due to the 5 years of data exclusivity awarded for all NMEs that are small-molecule drugs. There is a peak of generic entries in year 7 due to expirations of orphan data exclusivities for rare diseases as well as the expiration of the 7.5-year stay provision for drugs under patent challenge. The peak in year 14 is due to the expiration of patent term restoration, which has a 14-year maximum that is commonly reached.*

| **APPENDIX TABLE 2. Drugs approved by FDA after 01/01/1997** | | | | | | | |
| --- | --- | --- | --- | --- | --- | --- | --- |
| Drug | Post Approval Exclusivity Codes | Drug | Post Approval Exclusivity Codes | Drug | Post Approval Exclusivity Code | Drug | Post Approval Exclusivity Code |
| TEVETEN | - | NUVARING | - | FERRIPROX | - | ANTIZOL | ODE, I-319 |
| AVAPRO | - | EVOXAC | - | AMMONIA N 13 | - | CORLOPAM | I-422 |
| ATACAND | - | AXERT | - | UROXATRAL | - | HECTOROL | I-424 |
| MICARDIS | - | FROVA | - | LEVITRA | - | MOBIC | I-430, ODE |
| TIKOSYN | - | RELPAX | - | ENABLEX | - | ALDARA | I-433 , I-420 |
| BENICAR | - | RAZADYNE | - | RAPAFLO | - | LIPITOR | I-434 |
| RANEXA | - | STRATTERA | - | GANIRELIX ACETATE | - | VFEND | I-442 |
| BYSTOLIC | - | CAMPRAL | - | AUBAGIO | - | PROTONIX IV | I-444, I-337 |
| LETAIRIS | - | LUNESTA | - | AGRYLIN | - | ARIXTRA | I-457 |
| LIVALO | - | NAMENDA | - | FARESTON | - | CELEBREX | I-466 |
| ACIPHEX | - | AZILECT | - | VALSTAR PRESERVATIVE FREE | - | CYMBALTA | I-470, I-524, I-556 |
| LOTRONEX | - | ROZEREM | - | ARAVA | - | TARCEVA | I-473 |
| ELLA | - | BANZEL | - | TEMODAR | - | EMEND | I-475 |
| EPIVIR | - | INVEGA | - | TARGRETIN | - | INVANZ | I-476, I-515 |
| SUSTIVA | - | FANAPT | - | TRISENOX | - | TRILEPTAL | I-478 |
| ZIAGEN | - | AMPYRA | - | ELOXATIN | - | FEMARA | I-481, I-446, I-318 |
| FACTIVE | - | SAVELLA | - | LOVAZA | - | ANGIOMAX | I-486, I-458 |
| SPECTRACEF | - | VIIBRYD | - | CLOLAR | - | ZETIA | I-493 |
| KALETRA | - | PRANDIN | - | DACOGEN | - | AROMASIN | I-495 |
| VIREAD | - | AVANDIA | - | TORISEL | - | PLAVIX | I-502 |
| HEPSERA | - | ACTOS | - | GILENYA | - | KEPPRA | I-506, I-527, I-563 |
| EMTRIVA | - | ORFADIN | - | ELLENCE | - | YAZ | I-508, I-522 |
| MYCAMINE | - | INOMAX | - | VIDAZA | - | ARICEPT | I-529 |
| REYATAZ | - | ZADITOR | - | BUTAZOLIDIN | - | EVISTA | I-539, I-271, ODE |
| CUBICIN RF | - | CLARINEX | - | ZIOPTAN | - | WELCHOL | I-553, I-606 |
| TINDAMAX | - | BEPREVE | - | ALREX | - | AVODART | I-565 |
| BARACLUDE | - | DALIRESP | - | TRAVATAN | - | QUETIAPINE Fumarate | I-574, I-575, I-576 |
| PREZISTA | - | ELESTAT | - | LUMIGAN | - | PRECEDEX | I-577 |
| NOXAFIL | - | TAZORAC | - | LOTEMAX | - | TYGACIL | I-586 |
| OMNICEF | - | ABREVA | - | DOTAREM | - | ZYPREXA | I-591 |
| VESICARE | - | ELIDEL | - | FOSRENOL | - | ZEMPLAR | I-599 |
| SANCTURA | - | ALTABAX | - | ABLAVAR | - | TRACLEER | I-607 |
| TOVIAZ | - | ZAVESCA | - | XYREM | ODE, ODE-231 | AFINITOR | I-638, ODE |
| BONIVA | - | ALOXI | - | SPRYCEL | ODE-225 | CIALIS | I-641 |
| ULORIC | - | ENTEREG | - | ABILIFY | ODE-80 | LYRICA | I-651 |
| SABRIL | - | KUVAN | - | RECLAST | I-271, I-581, I-584, I-595 | ELIQUIS | I-661 |
| GABITRIL | - | INTEGRILIN | - | ACTONEL | I-309 | PRISTIQ | I-675 |
| REQUIP | - | PLETAL | - | TAMIFLU | I-317, I-480 | KYPROLIS | I-722 |
| MIRAPEX | - | FERRLECIT | - | XELODA | I-323, I-341, I_323 | FASLODEX | I-725, I-749 |
| TASMAR | - | REMODULIN | - | AVELOX | I-329, I-385, I-447, I-479 | CRESTOR | I-732, ODE |
| PROVIGIL | - | FIRAZYR | - | PROTONIX | I-330, I-356 | AFINITOR DISPERZ | I-773, ODE-169 |
| AMERGE | - | EFFIENT | - | ARGATROBAN | I-352 | MYRBETRIQ | I-777 |
| ZOMIG | - | BRILINTA | - | ZOMETA | I-361, I-581 | EXJADE | ODE |
| ZONEGRAN | - | PRADAXA | - | SINGULAIR | I-378, I-465, I-530 | TOPAMAX | ODE |
| COMTAN | - | INSPRA | - | GLEEVEC | I-392, I-512, I-666, ODE | COLAZAL | ODE |
| CELEXA | - | FLOMAX | - | CANCIDAS | I-399 | SENSIPAR | ODE |
| EXELON | - | MIFEPREX | - | ZYVOX | I-402, I-431 |  |  |
| GEODON | - | DETROL | - | STARLIX | I-411 |  |  |
| SONATA | - | VIAGRA | - | SEROQUEL | I-419, I-574 |  |  |
| SEPTOCAINE |  | ORTHO EVRA |  |  |  |  |  |

| **APPENDIX TABLE 3. Number of Indications at the time of approval** | |  |
| --- | --- | --- |
| Number of Indications | Number of Drugs |  |
| 1 | 163 |  |
| 2 | 22 |  |
| 3 or more | 12 |  |
| The indications are extracted from the FDA Approval Letter and Packaging label for each drug. The Approval letters and the packaging labels are available from: https://www.accessdata.fda.gov/ | |  |
|  |  |  |
|  |  |  |
| REFERENCE:Approved Drugs.Drugs@FDA. USA Food and Drug Administration. Available from: https://www.accessdata.fda.gov/. Accessed 11 Oct 2020 | |  |
|  |  |  |
